# Deep learning models for cancer stem cell detection: a brief review

**DOI:** 10.3389/fimmu.2023.1214425

**Published:** 2023-06-27

**Authors:** Jingchun Chen, Lingyun Xu, Xindi Li, Seungman Park

**Affiliations:** ^1^ Nevada Institute for Personalized Medicine, University of Nevada, Las Vegas, Las Vegas, NV, United States; ^2^ School of Life Science and Technology, Wuhan Polytechnic University, Wuhan, China; ^3^ Department of Mechanical Engineering, University of Nevada, Las Vegas, Las Vegas, NV, United States

**Keywords:** cancer stem cells (CSCs), artificial intelligence (AI), deep learning, convolutional neural network (CNN), image classification

## Abstract

Cancer stem cells (CSCs), also known as tumor-initiating cells (TICs), are a subset of tumor cells that persist within tumors as a distinct population. They drive tumor initiation, relapse, and metastasis through self-renewal and differentiation into multiple cell types, similar to typical stem cell processes. Despite their importance, the morphological features of CSCs have been poorly understood. Recent advances in artificial intelligence (AI) technology have provided automated recognition of biological images of various stem cells, including CSCs, leading to a surge in deep learning research in this field. This mini-review explores the emerging trend of deep learning research in the field of CSCs. It introduces diverse convolutional neural network (CNN)-based deep learning models for stem cell research and discusses the application of deep learning for CSC research. Finally, it provides perspectives and limitations in the field of deep learning-based stem cell research.

## 1 Introduction

**Figure 1:**
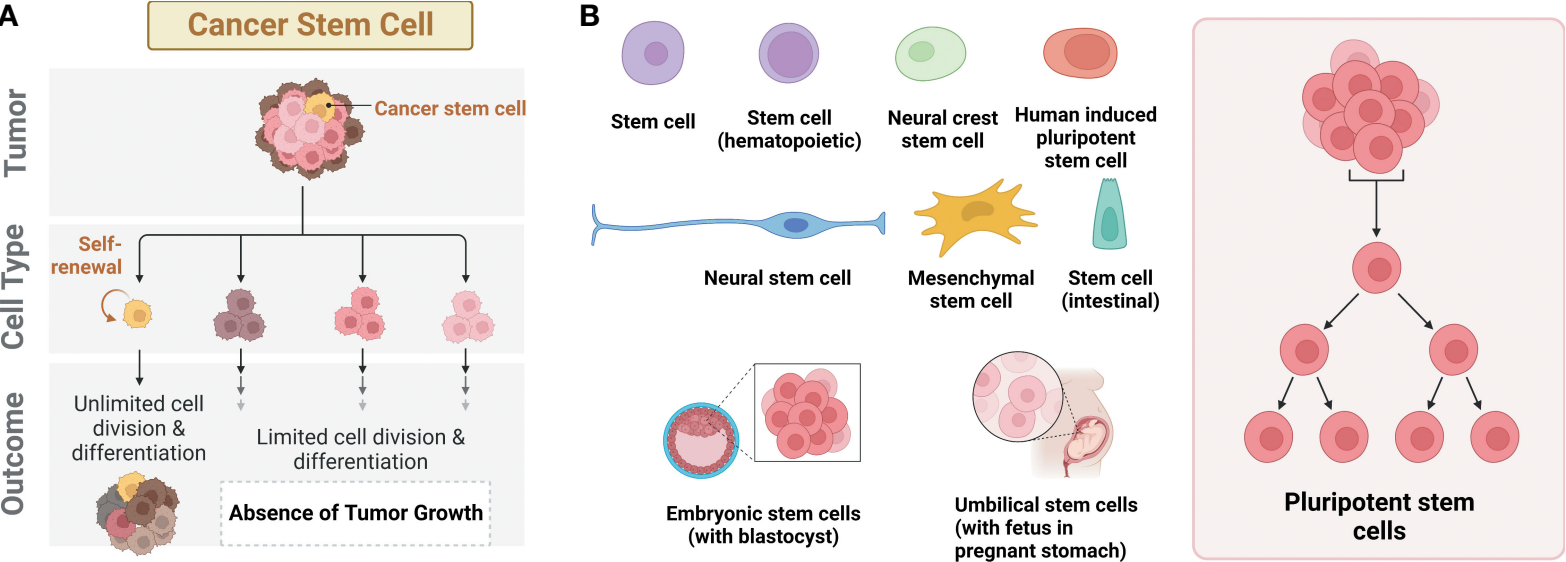
Different types of stem cells. **(A)** Cancer stem cells (CSCs) with the ability to form tumors. **(B)** Different types of stem cells reported with deep learning AI technology.

Cancer stem cells (CSCs), also known as tumor-initiating cells (TICs), are a subpopulation of tumor cells. (1). These cells are thought to persist within tumors as a distinct population, driving tumor initiation, relapse, and metastasis through self-renewal and differentiation into multiple cell types, similar to the typical stem cell processes (**Figure 1A**). CSCs have been observed in various solid tumors, such as lung, liver, breast, stomach, and colorectal cancer. In 1877, Cohnheim first observed a minor group of cells displaying an embryonic characteristic in the tumor cells. The term, CSC, was first named in a paper published by Reya et al. (2–4). Since 2001, CSC has been one of the crucial interests in the field of cancer biology, diagnosis, and treatments of cancer patients.

It is widely accepted that CSCs are highly resistant to chemotherapy, and thus intensely involved in tumor relapses, as compared to well-differentiated or differentiating tumor cells (5, 6). Currently, the most challenging part about CSCs is how to accurately classify and separate them from tumor cells, due to their significant similarity to typical stem cells or tumor cells. One of the effective ways is to utilize the expression of several known surface markers, such as CD24, CD29, CD44, CD90, CD133, aldehyde dehydrogenase 1 (ALDH1), and epithelial-specific antigen (ESA). Those surface markers can be a therapeutic, diagnostic, or prognostic target for CSCs (7–10). However, some of the markers, such as CD133 or CD44, are commonly expressed in both CSCs and normal stem cells, which may lead to misdiagnosis (11). Thus, finding unique functional markers for CSCs or stem cells is essential to classify CSCs from non-CSCs and other types of stem cells.

One of those potential biomarkers is the morphology features, including cell/organelle morphology, that can often be measured qualitatively and quantitatively. Studies have shown that CSCs have different cell sizes with a different cytoplasmic to nuclear ratio and colony borders, as compared to non-stem cells (12). However, no standardized method has been established to differentiate CSCs from other cell types based on morphological features (1, 13). Furthermore, some types of stem cells, such as induced pluripotent stem cells (iPSCs), are found to be morphologically similar to CSCs. Thus, it is essential to develop a robust method not only for this purpose but also eventually for improving our understanding of cancer biology.

Recent remarkable advances in artificial intelligence (AI) technology enable automated recognition of various biological images for stem cell research. The majority of this research has been focused on classifying the different states of stem cells, such as colonies, single cells, differentiated cells, and dead cells, using different types of stem cells including iPSC, embryonic stem cells (ESCs), hematopoietic stem cells (HSCs), mesenchymal stem cells (MSCs), adult stem cells (ASCs), and neural stem cells (NSCs) (**Figure 1B**). In contrast, only a few studies have been focused on CSCs with the application of AI, especially deep learning. In this mini-review, we address a recent trend for deep learning research using CSCs. First, diverse convolutional neural network (CNN)-based deep learning models for stem cell research are introduced. Next, the application of deep learning for CSC research is discussed. Lastly, we provide perspectives and limitations in the field of deep learning-based stem cell research.

## 2 Deep learning for stem cell research

### 2.1 CNN-based deep learning models for stem cell research

CNN is a type of deep neural network that is primarily employed in image classification and object recognition based on the correlation of neighboring pixels. Like most deep machine learning, CNN is composed of three steps (training, testing, and validation). Each step consists of a multilayer neural network starting with randomly defined patches for input and modifying them during the training process. Once training is done, the network uses these modified patches to predict and validate the result in the testing and validation process.

Stem cells are defined as primitive or undifferentiated precursor cells with the ability to perform self-renewal division and differentiation into diverse mature and functional cell types of an organism. In general, stem cells are classified into three categories: ESCs, ASCs, and iPSCs (14). ESCs are pluripotent stem cells that can differentiate into all derivatives of the ectoderm, endoderm, and mesoderm germ cell layers that consist of lineage-specific stem cells, such as HSCs, MSCs, and NSCs. ASCs are multipotent stem cells that can develop into multiple specialized cells in a specific tissue, such as blood cells, skin, bone, cartilage, and cardiac muscles (15). HSCs, an example of ASCs, are derived from ESCs but have less differentiation potency than ESCs. Lastly, iPSCs are developed by genetically reprogramming mature cells, such as human somatic cells, into embryonic-like stem cells (16).

Stem cells have the power to differentiate multiple or specific functional cells or tissues; thus, studying stem cells may help explain the mechanisms and development process underlying human normal developmental physiology and disease pathology. Stem cells hold to have great potential for the future of cell-based therapeutic approaches in the field of regenerative medicine (17), precision medicine (18, 19), and cancer therapy (20). Examples include Parkinson’s disease, Alzheimer’s disease (21), spinal cord injury (22), heart disease (23), diabetes (24), and arthritis (25). Stem cells can also be used for drug screening and drug discovery (26). However, before its applications in clinical practice, it is essential to characterize stem cells and assess their differentiation potential and functionality.

Traditional characterization of stem cells relies on the manual analysis of specific molecular techniques, such as immunostaining with specific antibodies or lineage tracing. Such analysis of large datasets is time-consuming, error-prone, and training-dependent (27). Each cell type shows a distinct characteristic morphology based on cell type-specific gene expression. Although we cannot identify cell type-specific morphology by microscopic observation alone, an automated morphology-based identification system by CNN have been developed to classify label-free stem cells at different differentiation status in the last decades.

Nowadays, CNN as a high-throughput imaging method has applied to relevant cell biology with different cell culture models, fostering its use in research and translational applications. Improvements in microscopy, computational capabilities, and data analysis have enabled high-throughput, high-content approaches from endpoint 2D microscopy images to 3D microscopy images. This approach has been engaged in various cell biology research activities (28–30), specifically for stem cell characterization (31–33) and differentiation pattern (34–37), disease modeling (38, 39), and drug screening and discovery (40, 41). The advantages of this technique are that it is non-invasive, high-throughput, and consistent, which can save time and costs once automated.

The most common CNN techniques include VGG19, InceptionV3, Xception, ResNet50V2, and DenseNet121 (42–44). Kim et al.’s study demonstrated that DenseNet121 was the most promising model with an area under the curve (AUC) of 0.975 and an accuracy of 0.922 when they screened mesenchymal stem cell lines with deep learning (44). Park et al. later confirmed that DenseNet121 was the best model to predict the differentiation of kidney organoids. Kegeles et al. developed a deep learning-based computer algorithm to recognize retinal differentiation in stem cell-derived organoids based on brightfield imaging. In the study, they compared different learning approaches: ImageNet pre-trained ResNet50v2, VGG19, Xception, and DenseNet121 CNNs. As a result, they found that ResNet50v2 was the best-performing classifier with an AUC of 0.91, which performed better than the expert in predicting organoid fate (42). Additionally, Schaub et al. developed a robust characterization methodology integrating quantitative brightfield absorbance microscopy (QBAM) and deep neural networks (DNNs) to predict tissue function of live retinal pigment epithelium, demonstrating non-invasive cell therapy characterization could be achieved with QBAM and machine learning (31). Later, Dursun et al. developed CNNs for the recognition of tenogenic differentiation and found that Inception-ResNetV2 was the best model in terms of accuracy of 96.80% and training time of 434.55 sec (32).

Nevertheless, many challenges arise in the real-time process. For example, low-quality micrographs are often obtained due to technical issues during the experiment. To improve the performance with those low-quality images, Sun et al. developed a cell image-enhanced generative adversarial network (CIEGAN) for image enhancement. They applied the CIEGAN to long-term live-cell imaging of a human-induced pluripotent stem cell-derived cardiomyocyte (hiPSC-CM) differentiation system, which greatly enhanced the quality of brightfield cell images from 256 by 256 pixels to 1,024 by 1,024 pixels, which was 16 times the quantity of pixels (35).

### 2.2 Application of deep learning on CSCs

Currently, many studies have been reported to apply deep learning to stem cell research for the identification of stem cell differentiation, reprogramming, fate, and multipotency (**Table 1**). For example, Zhu et al. applied a deep learning model (Xception) to bright field images (149,428 images) to investigate the fate of primary NSCs derived from rats. To collect data for a model system, they created and used NSCs in different phases with differentiation inducers: neurons, astrocytes, and oligodendrocytes. Notably, the model was able to provide an accurate prediction of cell differentiation with a single-cell image even after NSCs were co-cultured for 1 day with the inducers (45). Waisman et al. adopted different CNN models (ResNet34, ResNet50, ResNet101, and DenseNet) to distinguish PSCs from early differentiating cells. Transmitted light microscopy images for the induced differentiation of mouse ESCs to epiblast-like cells were utilized. Results showed that the used models had a good capability to identify undifferentiated and differentiating cells with high accuracy (> 99%) (46). Fan et al. leveraged a label-free and non-invasive brightfield imaging analysis system combined with deep learning (modified AlexNet) to determine iPSC colony formation (47). Kavitha et al. developed a new model, vector-based CNN (V-CNN), to distinguish iPSC colonies with phase contrast images and their results were compared with a support vector machine (SVM) classifier. The accuracy of the V-CNN model (> 90%) was significantly higher than that (75-77%) of the SVM model (48). Schaub et al. proposed a new non-invasive approach consisting of quantitative brightfield absorbance microscopy and a CNN-based deep learning model to predict stem cell donors and tissue functions (31). For the validation, QBAM images of iPSC-RPE were tested by training the modified GooglNet for the prediction of iPSC-RPE monolayer transepithelial resistance, polarized vascular endothelial growth factor secretion, and the stem cell donors by matching iPSC-RPE monolayers and their performance was compared with L-SVM. Results exhibited that the deep learning model has better performance with an accuracy of 85.4%, a sensitivity of 80.9%, and a specificity of 86.8%, compared to L-VSM with an accuracy of 76.4%, a sensitivity of 64.6%, and a specificity of 82.3%. Kim et al. proposed a new prediction method with a CNN model (DenseNet121) to evaluate the multipotency rate of Human nasal turbinate stem cells (hNTSCs) using fluorescence images by characterizing genes and morphologies. The CNN model classified multipotent cells comparatively well with 85.98% accuracy, which well-matched results of actual differentiation (33).

**Table 1:** Representative deep learning studies that have been conducted for the detection and classification of different types of stem cells.

AI (CNN) model	Image Type	Application	Cell Type/Source	References
Xception (Upgrade version of Google Inception)	Brightfield	NSC fate identification	Primary neural stem cell from rat	(45)
ResNet34, ResNet50, ResNet101, DenseNet	Brightfield	PSC differentiation	Mouse ESCs and human iPSCs	(46)
Modified AlexNet	Brightfield	iPSC colony formation for studying cell reprogramming	Mouse embryonic fibroblast and human urinal cell	(47)
Deep vector-based CNN	Phase contrast	iPSC colony detection	Murine embryonic fibroblast and iPSC line	(48)
Modified GoogleNet	Brightfield absorbance images	Prediction of iPSC-RPE function	hiPSC-derived retinal pigment epithelial cells	(31)
DenseNet121	Fluorescence	Prediction of human stem cell multipotency	Human nasal turbinate stem cells (hNTSCs)	(33)
Homemade-deep learning-based algorithms (DLBAs) with 4 convolution layers	Brightfield	CSC fate detection	Glioblastoma-derived N14-0510 and N14-1525 CSCs	(49)
Conditional generative adversarial networks (CGAN), ResNet50, VGG16	Phase contrast	CSC segmentation	miPS-LLCcm * CSCs	(1, 50)

*miPS-LLCcm: A mouse-induced pluripotent stem (miPS) cell cultured in a medium containing Lewis lung cancer (LLC) cell culture-conditioned medium (cm).

Nevertheless, the studies and data for AI applications in CSCs are limited. One of the most significant barriers is that little is known about the characteristic features of CSC morphology (51). Previous research has shown that CSCs are phenotypically transformed from non-CSCs (50). One study showed that iPSC-induced CSCs (i.e., malignant cells with stem-like properties) formed spheroids under non-adherent conditions, whereas non-CSCs (fibroblast-like cells) did not survive. The study indicated significant changes in morphology during the transformation from non-CSCs to CSCs (13). Further studies found that iPSC-derived CSCs with gene *Nanog* expression formed spherical colonies, but CSCs without *Nanog* expression didn’t (13, 52). However, other studies showed that there was no difference in morphology between CSCs and non-CSCs. For example, human nasopharyngeal carcinoma-derived CSCs and non-CSCs displayed the same squamous morphology, while the CSCs showed morphological changes in different culture media and culture periods (53). With a few studies about the morphological features of CSCs, it is still challenging to accurately identify and determine their characteristics by experts or trainees in the field of stem cells. Generally, AI may improve the experts’ performance in the classification of CSCs (46, 51). To our knowledge, only a few papers were reported from a small number of groups regarding deep learning and CSC detection.

From large amounts of brightfield images, Chambost et al. developed and utilized a deep learning-based algorithm (DLBAs) with 4 convolutional layers for label-free and real-time detection of CSC fate and state (division, death, or quiescence) (49). The features of this model encompass 4 convolutional and 3 max-pooling layers, which are followed by a first fully connected layer. Additionally, there is a second fully connected layer responsible for classifying images such as “Singles,” “Multiples,” “Death,” or “Empty.” The results demonstrated that the DLBA had a better performance with an accuracy of 91.2% and computation time of 0.02 s per image when compared with a shallow learning-based algorithm (SLBA) and several CNN-based models (classical CNN, ResNet50, Inception V3, and VGG16).

Hanai et al. adopted an AI model based on an image-to-image translational system called conditional generative adversarial networks (CGAN) (54) to identify live CSC morphology in phase-contrast images (50). CGANs learn a loss function aimed at identifying and distinguishing between real and fake output images, while concurrently training a conditional generative model to minimize this loss. This characteristic renders cGANs highly suitable for image-to-image translation tasks (54, 55). They induced CSCs by culturing a mouse-induced pluripotent stem (miPS) cell in a medium containing Lewis lung cancer (LLC) cell culture-conditioned medium (cm) - miPS-LLCcm. Further information on the induction method is reported elsewhere (13). Cell image datasets were obtained from cultured plates on days 1 and 2. Results showed that the classification performance for CSC was better at an earlier stage of cell culture, but became worse with more days in cultures. Thus they utilized images from the 1-day culture for training and the 2-day culture for testing. Furthermore, they found that there was a high precision of CSC classification for both ResNet50 (0.888 from the 1-day dataset and 0.946 from the 2-day dataset) and VGG16 models (0.862 from the 1-day dataset and 0.934 for the 2-day dataset) when they classified CSCs *via* transfer learning with the ResNet50 and VGG16 models. To further improve classification, they used F-measure datasets for CGAN image translation to understand the effect of differences in phase-contrast images on the depiction of CSCs. As a result, they found several more accurate AI models (1). These studies proved that these deep learning models could be versatile in distinguishing undefined morphological features in CSCs.

## 3 Discussion

There are several advantages to using pre-trained CNN models instead of retraining AI models from scratch in the context of CSC research (56, 57). First, CSC data is extremely limited and scarce compared to general stem cell data (51), making it challenging to train AI models solely on CSC data. Pre-trained models offer a way to leverage knowledge acquired from large and diverse datasets, effectively utilizing existing knowledge to enhance performance even with limited CSC data. Second, pre-trained CNN models have already learned features and patterns from extensive amounts of data, enabling them to generalize well to new and unexplored CSC data. They can capture common visual patterns and relationships that are relevant to CSC analysis, resulting in improved performance, as compared to training models from scratch, which may require a more extensive dataset for effective generalization. Last but not least, training deep learning models from scratch can be computationally expensive and time-consuming. By utilizing pre-trained models, the initial training phase has already been completed, reducing computational costs and expediting the overall training process. However, it should be noted that the effectiveness of pre-trained models compared to training from scratch may vary, depending on the specific characteristics of the CSC data or the availability of suitable pre-trained CNN models. Therefore, conducting comprehensive experiments and comparisons is crucial to determine the most effective approach for a given CSC-related study.

Despite significant advancements in AI technology and its implementation in stem cell research, including CSCs, there remain several obstacles that must be addressed (58, 59). First, although several models have been so far developed, most of the studies only chose one or a few models and compared the performances with accuracy or precision. Without testing other powerful models, such designs could not guarantee that the selected models would produce the best performance among currently developed models. To overcome this limitation, Park’s group developed a global deep learning model system by performing approximately 20 widely used CNN-based deep learning models. After testing those well-known models, they chose and combined five models with the top accuracy to create a global model. As a result, their performance was improved significantly, as compared to that of individual models (60).

Another challenge is that the morphological features of CSCs remain elusive (1). Currently, it is difficult to define and generalize the morphological properties of CSCs because their morphology is highly dependent on cell type, culture condition, etc. Moreover, stem cells are highly dynamic and heterogeneous in that they have a variety of shapes, ranging from spherical to irregular, spindle-shaped, or elongated. This complexity makes it challenging to design and develop deep learning models that can accurately capture the relevant features of CSCs. Mor et al. observed that ovarian cancer stem cells (Type I Epithelial ovarian cancer (EOC) stem cells) were bigger and had a higher nucleus-to-cytoplasm ratio with vesicular chromatin pattern and prominent nucleoli, as compared to ovarian cancer cells (Type II EOC cells) (61). Another experimental investigation by Zhang et al. revealed that colorectal CSCs formed a sphere in the normal culture medium for 10 days, but CSCs changed to adherent cells after the serum was added to the culture medium (62). Further studies should be warranted to investigate and define what exact morphological characteristics represent CSCs.

Deep learning models are often considered black boxes, making it challenging to interpret how and why they make specific predictions. This lack of interpretability can be problematic in CSC research, as it is crucial to understand the underlying biological mechanisms and identify key features for decision-making. As such, interpretability is vital for researchers to gain insights into the biology of CSCs and guide further experimental studies. To enhance interpretability, intermediate layers of a CNN can be visualized. The visualization of intermediate layers can indeed provide some interpretability of the results (63–65). By visualizing the activations or feature maps of intermediate layers, we can gain insights into what specific patterns or features the network is learning and detecting at different stages of the model. The intermediate layer visualization helps us understand how information is transformed and processed within the network. By observing the activations, we can identify which regions of the input image contribute more strongly to the final output. This can provide valuable insights into the decision-making process of CNN and help interpret the results. Furthermore, visualizing intermediate layers can also help detect issues like overfitting or underfitting. If the activations appear too sparse or too concentrated, it may indicate a problem in the learning process of the modeling. By examining the visualization, researchers can gain a better understanding of how CNN processes the data and potentially identify areas for improvement or optimization. However, it is important to note that while intermediate layer visualization can provide valuable interpretability, it is not a foolproof method for understanding the entire decision-making process of a complex CNN. Thus. interpretability in deep learning models remains an ongoing research area, and multiple techniques are often used to improve understanding and explainability in conjunction with layer visualization.

With deep learning algorithms, the accuracy of cell classification and segmentation heavily relies on the quality of the captured images (66). Most errors observed during the training and testing phases can be attributed to inadequate image contrast and regions with blurry image features. As such, high contrast and visible images should be obtained and used to distinguish stem cell dynamics and morphology. Currently, phase-contrast or brightfield microscopy has been widely used to quantify cellular morphological characteristics and evaluate cellular phenotypes as a non-invasive method. However, in some cases, it is challenging to take contrast images with high quality, as microscopy generally exhibits high sample-to-sample variability. As an alternative, fluorescence microscopy can be leveraged to provide better images for visualization by labeling cells with fluorescence dyes. One of the limitations of fluorescence dyes is that they may affect or damage cell organelles, functions, or behaviors due to toxic compounds, phototoxicity, and biochemical artifacts. If we need to use the cells in their downstream study, this labeling would not be the first choice. Moreover, cell labeling is time-consuming and expensive. Overall, large, high-quality datasets are required to effectively train different models with deep learning in different kinds of CSCs. Currently, data are limited in the relatively new field for stem cell research, (67).

In addition to that, validating the performance and generalizability of deep learning models is essential before their clinical applications. In particular, it is essential to ensure that the trained models can accurately identify and classify CSCs in different experimental settings and diverse patient populations. Performing robust validation studies with large and various datasets is crucial to assess the reliability and generalizability of these models.

The integration of expert experience or knowledge in deep learning models has the potential to significantly enhance their overall effectiveness and generalization ability (68). By incorporating the knowledge and expertise of domain experts, CNN models can benefit from a deeper understanding of the specific problem or domain they are applied to. This integration can result in improvements in various aspects such as feature extraction, architecture design, hyperparameter tuning, and data labeling and annotation. Moreover, expert experience provides valuable insights into the intricate nuances of the data, enabling more accurate predictions. Consequently, the integration of expert experience into CNN models holds the promising potential to elevate their effectiveness and generate more reliable results.

In conclusion, advanced deep learning techniques combined with high-resolution imaging modalities and expert experience will provide us with the precise identification and classification of CSCs and speed up our understanding of CSCs’ functional properties in an automatic way.

## Author contributions

SP and JC reviewed the conception and design, drafted the manuscript, critical revisions, and addressed the reviewers’ comments. LX and XL conducted a literature search and summarized the literature. All authors contributed to the article and approved the submitted version.
